# Identification of the SARS-CoV-2 Delta variant C22995A using a high-resolution melting curve RT-FRET-PCR

**DOI:** 10.1080/22221751.2021.2007738

**Published:** 2021-12-21

**Authors:** Subarna Barua, Jianfa Bai, Patrick John Kelly, Gregg Hanzlicek, Lance Noll, Calvin Johnson, Ji-Hang Yin, Chengming Wang

**Affiliations:** a College of Veterinary Medicine, Auburn University, Auburn, AL, USA; b Kansas State Veterinary Diagnostic Laboratory, College of Veterinary Medicine, Kansas State University, Manhattan, KS, USA; c Department of Veterinary Clinical Sciences, Ross University School of Veterinary Medicine, St. Kitts & Nevis

**Keywords:** FRET-PCR, SARS-CoV-2 Delta variant, C22995A mutation, high-resolution melting curve analysis, diagnostic laboratories

## Abstract

Knowledge of SARS-CoV-2 variants is essential for formulating effective control policies. Currently, variants are only identified in relatively small percentages of cases as the required genome sequencing is expensive, time-consuming, and not always available. In countries with facilities to sequence the SARS-CoV-2, the Delta variant currently predominates. Elsewhere, the prevalence of the Delta variant is unclear. To avoid the need for sequencing, we investigated a RT-FRET-PCR that could detect all SARS-CoV-2 strains and simultaneously identify the Delta variant. The established Delta RT-FRET-PCR was performed on reference SARS-CoV-2 strains, and human nasal swab samples positive for the Delta and non-Delta strains. The Delta RT-FRET-PCR established in this study detected as few as ten copies of the DNA target and 100 copies of RNA target per reaction. Melting points of products obtained with SARS-CoV-2 Delta variants (around 56.1°C) were consistently higher than products obtained with non-Delta strains (around 52.5°C). The Delta RT-FRET-PCR can be used to diagnose COVID-19 patients and simultaneously identify if they are infected with the Delta variant. The Delta RT-FRET-PCR can be performed with all major thermocycler brands meaning data on Delta variant can now be readily generated in diagnostic laboratories worldwide.

## Introduction

Detection of SARS-CoV-2 variants is essential for monitoring the COVID-19 pandemic and developing appropriate control policies [1]. Detecting variants, however, requires genome sequencing which is expensive and time-consuming and thus only infrequently performed [2]. This is the case even in developed countries where, for example, under 1% of positive cases in the US are sequenced [3]. We recently reported a reverse transcription fluorescence resonance energy transfer-polymerase chain reaction (RT-FRET-PCR) that can be performed with all major brands of PCR machines to detect mutations in the SARS-CoV-2 strains rapidly and conveniently [3]. Here, we establish a Delta RT-FRET-PCR that can be readily used by diagnostic laboratories around the world to detect SARS-CoV-2 infections and, simultaneously, the presence of the Delta variant (B.1.617.2).

## Method

Analysis of the available whole-genome SARS-CoV-2 sequences in GISAID (www.gisaid.org) confirmed previous reports that the C22995A (T478K) mutation is one of the most common mutations present in the SARS-CoV-2 Delta variant [4–6]. The C22995A mutation is present in 99.73% (320,730 / 321,061) of the Delta variant, but in only 0.006% (62 / 962,990) of classical isolates, three other variants of concern (VOC), and six variants of interest (VOI) (Table S1).

Also using the whole-genome SARS-CoV-2 sequences from GISAID, upstream and downstream primers were designed to target the spike gene with the amplicon size of 235 bp and amplify all SARS-CoV-2 strains we examined in GISAID. The 6-carboxyfluorescein (6-FAM)-labeled probe was specifically designed to contain the unique C22995A mutation: upstream primer: 5′-CAGGCTGCGTTATAGCTT-3′; downstream primer: 5′-TATGGTTGGTAACCAACACC-3′; 6-Fam-probe: 5′-CCGGTAGCAAMCCTTGTAAT-6-FAM-3′; LCRed 640 probe: 5′-LCR640- GTGTTGAAKGWTTTAWTTGTTACTTT -phosphate-3′.

The Delta RT-FRET-PCR was performed on a Roche Light Cycler 480 II system (Roche Molecular Biochemicals, Indianapolis, IN) as described [3]. Thermal cycling was preceded by a 15-minute reverse transcription reaction at 55°C followed by 4 min incubation at 95°C. Thermal cycling consisted of 18 high-stringency step-down cycles followed by 30 relaxed-stringency fluorescence acquisition cycles. The 18 high-stringency step-down thermal cycles were 6 × 10 sec at 95°C, 10 sec at 70°C, 10 sec at 72°C; 9 × 10 sec at 95°C, 10 sec at 68°C, 10 sec at 72°C; 3 × 10 sec at 95°C, 10 sec at 66°C, 10 sec at 72°C. The relaxed-stringency fluorescence acquisition cycling consisted of 30 × 10 sec at 95°C, 10 sec at 55°C followed by fluorescence acquisition, and 30 sec at 72°C.

**Figure 1.**
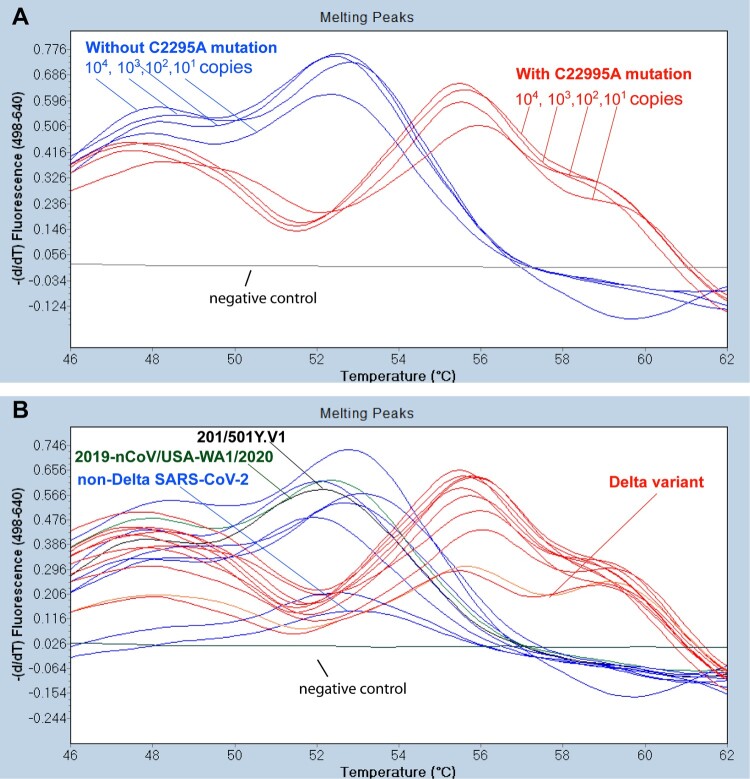
Differential melting temperatures of PCR products with non-Delta SARS-CoV-2 and the Delta variant. (A) With the 6-FAM probe designed to match the area incorporating the C22995A mutation exactly, dilutions of a control SARS-CoV-2 Delta variant (positive control from Kansas State Veterinary Diagnostic Laboratory) used in the Delta RT-FRET-PCR had a *T*_m_ of around 56.1°C. With the SARS-CoV-2 strains that did not have the mutation (2019-nCOV/USA-WA1/2020), the A to C mismatch with the probe resulted in a lower *T*_m_ of around 52.5°C. The *T*_m_ values did not vary significantly with the copy number. The negative control was RNA from a human nasal swab negative for SARS-CoV-2 by routine diagnostic PCR. (B) *T*_m_ analysis of representative Delta RT FRET-PCR products from controls and human nasal swab samples with non-Delta variant SARS-CoV-2 (blue lines) and Delta variants (red lines), all confirmed by DNA sequencing. The *T*_m_ values for the non-Delta strains were around 52.5°C, while those of the Delta variant were clearly different at around 56.1°C.

The melting curve analysis that assessed the dissociation of the PCR product and the 6-FAM probe was determined by monitoring the fluorescence from 35°C to 75°C with a temperature transition rate of 0.2°C per second as described [3]. The first derivatives of F2/F1 were evaluated to determine the *T*_m_ of the probe (Figure 1). The PCR products of all tested samples and controls were sent to ELIM Biopharmaceuticals (Hayward, CA, USA) for DNA sequencing.

DNA standards for the quantitative analysis of the Delta RT-FRET-PCR were generated using the PCR products of the 2019-nCOV/USA-WA1/2020 (purchased from ATCC), and a Delta variant confirmed by whole-genome sequencing [provided by Kansas State Veterinary Diagnostic Laboratory (KSVDL), College of Veterinary Medicine, Kansas State University, USA]. The PCR products were purified with the QIAquick PCR purification kit (QIAGEN, Hilden, Germany) and quantified with the PicoGreen DNA fluorescence assay (Molecular Probes, Eugene, OR) to generate quantitative standards. Standards for RNA quantification analysis studies consisted of 10-fold dilutions of the Quantitative Synthetic SARS-CoV-2 RNA (ATCC, USA).

Samples used to evaluate the Delta RT-FRET-PCR consisted of a positive and negative control SARS-CoV-2 sample used during routine screening of samples at KSVDL. The lab also provided 30 human nasal swab samples collected in March 2021 that were positive in SARS-CoV-2 PCR tests. Seventeen of the 30 positive nasal swabs were found to be the Delta variant by whole genome sequencing, with the remainder not having the C22995A mutation. Finally, we also tested two non-Delta controls purchased from the ATCC (2019-nCOV/USA-WA1/2020; 201/501Y.V1).

Comparison of the melting temperatures between SARS-CoV-2 viruses with and without C22995A mutation was analyzed by the T test (Statistica, StatSoft, Tulsa, USA). Differences at *P* ≤ 0.05 were considered significant.

## Results

The Delta RT-FRET-PCR established in this study was very sensitive, detecting as few as ten copies of the DNA target and 100 copies of RNA target per reaction.

The Delta RT-FRET-PCR was positive for the positive control sample from KSVDL, the reference SARS-CoV-2 strains from ATCC, and the 30 PCR positive human nasal swab samples provided by KSVDL that had been confirmed positive by whole genome sequencing. When the Delta RT-FRET-PCR was performed on the seventeen nasal swab samples from KSVDL that were confirmed positive for the SARS-CoV-2 Delta variant, each had a *T*_m_ of 56.1°C (Figure 1). In contrast, when the Delta RT-FRET-PCR was performed on the SARS-CoV-2 nasal swab strains that were not the Delta variant, the mismatch between the 6-FAM-probe and the strains resulted in a distinctly lower *T*_m_ value of 52.5°C. The clear difference in the *T*_m_ between the Delta variant and non-Delta strains enabled the convenient differentiation of strains with and without the C22995A mutation. The *T*_m_ did not change with target copy numbers (Figure 1).

Due to the additional nucleotide mismatch between FRET probes and non-Delta SARS-CoV-2, the amplification fluorescence curves are less smooth in non-Delta SAR-CoV-2 than the Delta variant (Figure S1). However, the distinctive melting curves remain sharp for SARS-CoV-2 with and without C22995A mutations (Figure 1).

DNA sequencing of the Delta RT-FRET-PCR products verified the presence and absence of the C22995A mutation as indicated by melting curve analysis. The delta variant with C22995A mutation demonstrated significant higher melting temperatures than those of non-Delta strains (56.13 ± 0.27 SD vs. 52.50 ± 0.23 SD; *p*<10^−4^).

## Discussion

The SARS-CoV-2 Delta variant has recently been classified as a variant of concern (VOC) by Public Health England (PHE), the World Health Organization (WHO), and the U.S. Centers for Disease Control (CDC) (https://www.cdc.gov/coronavirus/2019-ncov/variants/variant-info.html). Since its first detection in India in December 2020, the Delta variant has been identified in more than 130 counties [7] (https://gvn.org/covid-19/delta-b-1-617-2/). It is more than twice as transmissible as the original strain of SARS-CoV-2, and has become the major variant in many countries worldwide where genome sequencing is available to determine its presence [8].

Data on the distribution and prevalence of the Delta variant in countries with limited access to genome sequencing would enable a more global picture of the variant to be developed. Similarly, more detailed data on the epidemiology of the Delta variant in developed countries would enable a finer-scale analysis of the dynamics and public health implications of the strain [9].

A limitation of using the RT-FRET-PCR is that the C22995A mutation which it detects is also present in other variants, but these are only very rarely reported in GISAID (0.006%; 62 / 962,990), and false-positive results would be expected to be very unusual in symptomatic people. The Delta variant is just one of several SARS-CoV-2 variants that have been prevalent, so ongoing monitoring of strains from around the world will still be necessary to detect the evolution of new variants [1]. However, new RT-FRET-PCRs can rapidly be designed for these to readily enable widespread and detailed monitoring of their spread, epidemiology and characteristics. The sequence data we used to develop the primers for the Delta RT-FRET-PCR indicate the assay will detect all SARS-CoV-2 strains based on high-quality sequence data recorded in GISAID. Although the Delta RT-FRET-PCR detected all SARS-COV-2 strains against which it was tested in our study, further studies are indicated to more precisely define the sensitivity and specificity of the test in the diagnosis of COVID-19.

In conclusion, the Delta-RT-FRET-PCR we describe above proved to be very sensitive in detecting all the SARS-CoV-2 strains we tested, while simultaneously identifying those that were the Delta variant. Further, it can readily be performed with the thermocyclers supplied by the major vendors which are widely used in COVID-19 diagnostic laboratories around the world. The Delta-RT-FRET-PCR could, then, add considerably to the available knowledge on the spread of the Delta variant around the world, and facilitate the development of public health intervention programmes to counter the COVID-19 pandemic.
